# Case report of a patient with unclassified tauopathy with molecular and neuropathological features of both progressive supranuclear palsy and corticobasal degeneration

**DOI:** 10.1186/s40478-023-01584-z

**Published:** 2023-06-01

**Authors:** Shunsuke Koga, Michael A. Metrick, Lawrence I. Golbe, Alessia Santambrogio, Minji Kim, Alexandra I. Soto-Beasley, Ronald L. Walton, Matthew C. Baker, Cristhoper Fernandez De Castro, Michael DeTure, David Russell, Bradford A. Navia, Christine Sandiego, Owen A. Ross, Michele Vendruscolo, Byron Caughey, Dennis W. Dickson

**Affiliations:** 1 Department of Neuroscience, Mayo Clinic, 4500 San Pablo Road, Jacksonville, FL 32224 USA; 2 LPVD, Rocky Mountain Laboratories, NIAID, NIH, Hamilton, MT USA; 3 Centre for Misfolding Diseases, Yusuf Hamied Department of Chemistry, Cambridge University, Cambridge, UK; 4 Department of Neurology, Rutgers Robert Wood Johnson Medical School, New Brunswick, NJ USA; 5 Department of Artificial Intelligence and Informatics Research, Mayo Clinic, Jacksonville, FL USA; 6 Institute for Neurodegenerative Disorders, Temple Medical Center, New Haven, CT USA; 7 Invicro, LLC, New Haven, CT USA; 8 APRINOIA Therapeutics, Cambridge, MA USA; 9 Department of Clinical Genomics, Mayo Clinic, Jacksonville, FL USA

**Keywords:** Progressive supranuclear palsy, Corticobasal degeneration, Tauopathy, Tufted astrocyte, Astrocytic plaque, Tau PET, Real-time quaking-induced conversion

## Abstract

Progressive supranuclear palsy (PSP) and corticobasal degeneration (CBD) are distinct clinicopathological subtypes of frontotemporal lobar degeneration. They both have atypical parkinsonism, and they usually have distinct clinical features. The most common clinical presentation of PSP is Richardson syndrome, and the most common presentation of CBD is corticobasal syndrome. In this report, we describe a patient with a five-year history of Richardson syndrome and a family history of PSP in her mother and sister. A tau PET scan (^18^F-APN-1607) revealed low-to-moderate uptake in the substantia nigra, globus pallidus, thalamus and posterior cortical areas, including temporal, parietal and occipital cortices. Neuropathological evaluation revealed widespread neuronal and glial tau pathology in cortical and subcortical structures, including tufted astrocytes in the motor cortex, striatum and midbrain tegmentum. The subthalamic nucleus had mild-to-moderate neuronal loss with globose neurofibrillary tangles, consistent with PSP. On the other hand, there were also astrocytic plaques, a pathological hallmark of CBD, in the neocortex and striatum. To further characterize the mixed pathology, we applied two machine learning-based diagnostic pipelines. These models suggested diagnoses of PSP and CBD depending on the brain region – PSP in the motor cortex and superior frontal gyrus and CBD in caudate nucleus. Western blots of insoluble tau from motor cortex showed a banding pattern consistent with mixed features of PSP and CBD, whereas tau from the superior frontal gyrus showed a pattern consistent with CBD. Real-time quaking-induced conversion (RT-QuIC) using brain homogenates from the motor cortex and superior frontal gyrus showed ThT maxima consistent with PSP, while reaction kinetics were consistent with CBD. There were no pathogenic variants in *MAPT* with whole genome sequencing. We conclude that this patient had an unclassified tauopathy and features of both PSP and CBD. The different pathologies in specific brain regions suggests caution in diagnosis of tauopathies with limited sampling.

**Supplementary Information:**

The online version contains supplementary material available at 10.1186/s40478-023-01584-z.

## Introduction

Progressive supranuclear palsy (PSP) and corticobasal degeneration (CBD) are 4-repeat (4R) tauopathies classified as distinct types of frontotemporal lobar degeneration (FTLD)-tau [26]. The most typical clinical manifestations of PSP are postural instability with frequent falls, axial rigidity and vertical gaze palsy, referred to as Richardson syndrome [14, 30]. Corticobasal syndrome is the most common clinical presentation of CBD and is characterized by asymmetrical rigidity and apraxia, cortical sensory loss, alien limb phenomena and myoclonus [2, 4, 21]. Although PSP and CBD share genetic risk factors [25] such as homozygosity for the *MAPT* H1 haplotype [15], nearly all cases are sporadic; however, FTLD-tau due to *MAPT* mutations can mimic PSP or CBD [10].

Both PSP and CBD are characterized by aggregates of hyperphosphorylated tau protein in neurons (i.e., pretangles and globose tangles), oligodendrocytes (i.e., coiled bodies) and astrocytes (i.e., tufted astrocytes and astrocytic plaques) in cortical and subcortical structures [7, 29]. Since the distribution of tau lesions and neuronal loss and gliosis is different between PSP and CBD, differential diagnosis is usually relatively straightforward [22]. The pathognomonic astrocytic lesion in PSP is the tufted astrocyte, characterized by the accumulation of hyperphosphorylated tau in proximal processes in astrocytes [13]. In CBD, the pathognomonic astrocytic lesion is the astrocytic plaque, characterized by hyperphosphorylated tau aggregates in distal astrocytic processes [9]. A recent study with single-nucleus ATAC sequencing revealed different molecular alterations in astrocytes between the two diseases [6]. For these reasons, tufted astrocytes and astrocytic plaques, respectively, are diagnostic hallmarks of each disease and theoretically do not coexist in a single brain [24]; however, patients with atypical PSP or CBD have been reported with both types of astrocytic lesions [16, 39].

Here, we report three patients from one family with autosomal dominant familial PSP, including the results of pathological, biochemical and genetic analyses of the index case. Although the pathological diagnosis was partially consistent with PSP, the index patient also had astrocytic plaques in the cerebral cortices and striatum, which is atypical for PSP. Biochemical analysis of tau protein by Western blotting and real-time quaking-induced conversion (RT-QuIC) revealed features of both PSP and CBD. Whole-genome sequencing did not identify any previously reported pathogenic mutations in *MAPT* and *LRRK2*, which have been reported as causes of familial PSP.

## Case presentation

### Case 1

**Fig. 1:**
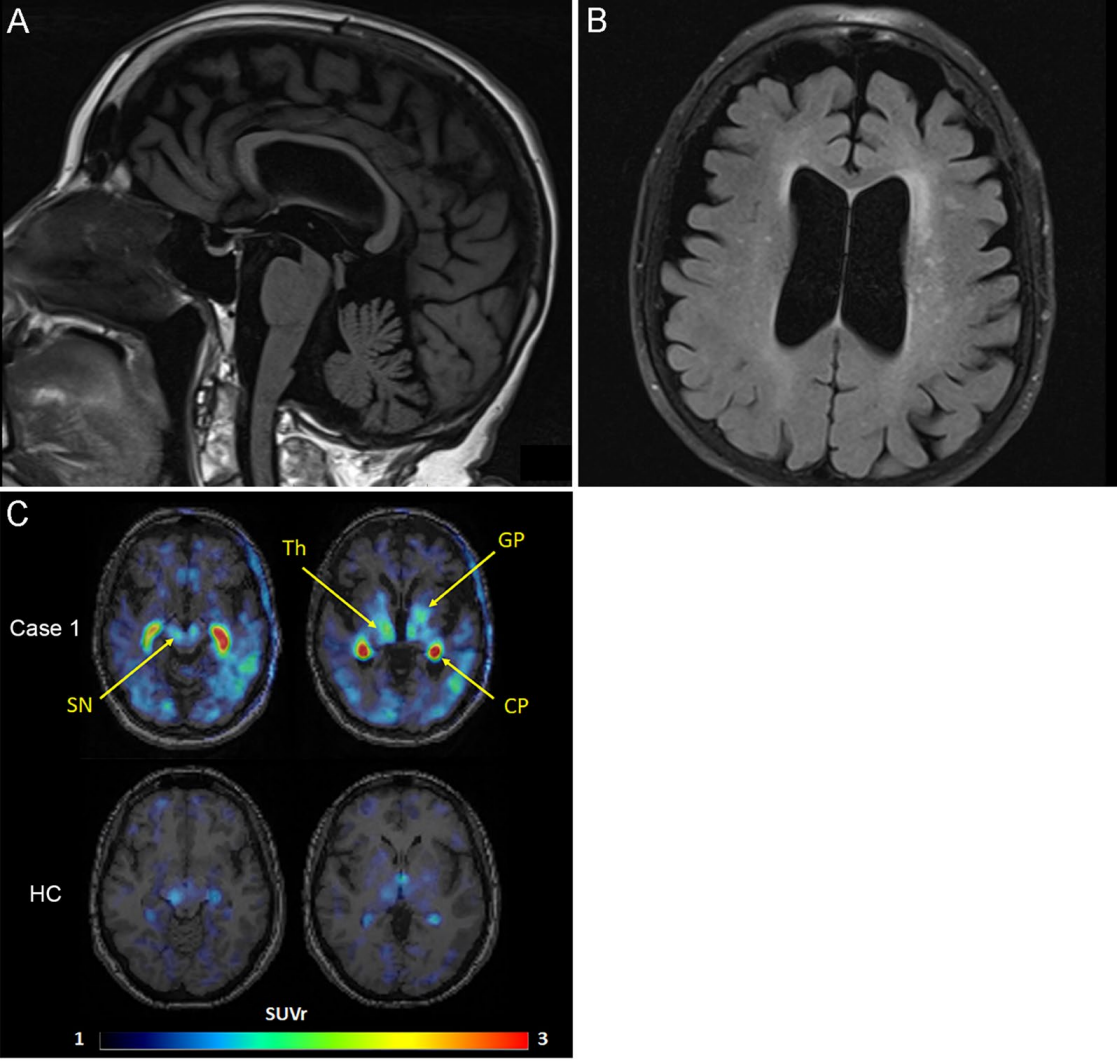
Representative images of brain MRI and tau PET. **A** The sagittal T1-weighted FLAIR image demonstrates atrophy of the midbrain. **B** The axial T2-FLAIR image displays asymmetrical cerebral atrophy with left-sided predominance, white matter hyperintensities, and dilation of the lateral ventricles. **C** Representative images of [^18^F]APN-1607 of Case 1 and a 54-year-old healthy female (HC). [^18^F]APN-1607 uptake is moderately increased in the globus pallidus, thalamus, and substantia nigra (indicated by arrows), compared to the HC. The two intense spots represent signal in choroid plexus (CP), which is non-specific and is occasionally seen in some patients and controls. The SUVr intensity color scale is shown at the bottom

The patient was a 75-year-old woman whose mother and sister had diagnoses of PSP. She presented with frequent falls at age 70, followed by bradykinesia. She developed urinary incontinence at age 72 and began having dysarthria, dysphagia, and diplopia at age 74. The neurological examination at age 74 revealed vertical gaze palsy in both directions, square wave jerks, mild spastic dysarthria, rigidity in the neck and extremities, bradykinesia, postural instability, and an unstable gait that required assistance. She also had a dystonic posture of left upper extremity and asymmetric motor difficulty worse on the left. Deep tendon reflexes were symmetric and within normal limits, but a Babinski sign was present on the left. No tremor, myoclonus, apraxia or orthostatic hypotension were observed. The total PSP Rating Scale score was 53 (Table 1) [12], typical for her symptom duration of 5 years. She scored 17/30 on the Montreal Cognitive Assessment, consistent with moderate cognitive impairment. Her deficits were characterized by impairments in visuospatial function, naming, attention, and delayed recall. The applause sign and antisaccade task were weakly abnormal. MRI of the brain revealed atrophy of the midbrain and mild, slightly asymmetric cerebral atrophy (Fig. 1A, B). An [^18^F]APN-1607 positron emission tomography (PET) scan revealed low-to-moderate uptake in a defined subset of brain structures, such as substantia nigra, globus pallidus, thalamus and posterior cortical areas, including temporal, parietal and occipital cortices (Fig. 1C). Low intensity signal was also noted across cortical regions, especially in the temporal lobes and posteriorly. Strong non-specific signal was also noted in choroid plexus, which is inconsistently seen with this tracer [38], but is not diagnostically significant. She was clinically diagnosed with probable PSP-Richardson syndrome.

**Table 1:** PSP rating scale of case 1

Items	Score	Interpretation
*I. History*		
1. Withdrawal	0	None
2. Aggressiveness	0	No increase in aggressiveness
3. Dysphagia	1	Tough food must be cut up into small pieces
4. Using knife and fork, buttoning clothes, washing hands and face	1	Somewhat slow but no help required
5. Falls	4	One or more per day (or chair bound)
6. Urinary incontinence	4	Consistent requiring diaper or catheter awake and asleep
7. Sleep difficulty	1	Either primary or secondary insomnia
*II. Mental Exam*		
8. Disorientation	3	Interfering with ADLs
9. Bradyphrenia	0	Clearly absent
10. Emotional incontinence	1	Equivocal or minimal
11. Grasping/Imitative/Utilizing behavior	1	Equivocal or minimal
*III. Bulbar Exam*		
12. Dysarthria	2	Definite moderate; most words comprehensible
13. Dysphagia	1	Fluid pools in mouth or pharynx or swallows slowly but no choking/coughing
*IV. Supranuclear Ocular Motor Exam*		
14. Voluntary Upward Saccades	4	15% of normal amplitude or worse
15. Voluntary Downward Saccades	1	Slow or hypometric; 86–100% of normal amplitude
16. Voluntary Left and Right Saccades	1	Slow or hypometric; 86–100% of normal amplitude
17. Eyelid Dysfunction	1	Blink rate decreased (< 15/minute) but no other abnormity
*V. Limb Exam*		
18. Limb Rigidity	3	Only partial range of motion possible
19. Limb Dystonia	3	Continuous but not disabling
20. Finger Tapping	1	Impaired (6–14 taps/5 s or moderate loss of amplitude)
21. Toe Tapping	2	Barely able to perform (0–5 taps/5 s or severe loss of amplitude)
22. Apraxia of Hand Movement	0	Absent
23. Tremor in Any Part	0	Absent
*VI. Gait I Midline Exam*		
24. Neck Rigidity or Dystonia	3	Only partial range of motion possible
25. Arising from Chair	4	Unable to arise without assistance
26. Gait	3	Must use assistance all or almost all of the time
27. Postural Stability (on backward pull)	4	Tends to fall without a pull; requires assistance to stand still
28. Sitting Down	4	Unable to test because of severe postural instability
*Total PSP Rating Scale*	53	

*ADL* activities of daily living

### Case 2 (sister of case 1)

This 71-year-old woman had a 6-year history of motor symptoms and personality changes. Her initial symptoms included difficulty waking, unsteadiness, frequent falls backward and blurred vision, as well as social withdrawal and memory problems. She did not have autonomic dysfunction, dream enactment behaviors or fluctuations in cognition. She was diagnosed with PSP by a neurologist at age 67. MRI of the brain at age 68 revealed atrophy of the superior cerebellar peduncle, but the midbrain was preserved. The same diagnosis was made by movement disorders specialists at three different institutions. The family history of PSP prompted genetic testing, which was negative for *C9ORF72*, *ANG*, *ARGHEF28*, *CDH13*, *CHMP2B*, *FUS*, *GRN*, *HNRNPA1*, *PSEN1*, *PSEN2*, *SOC1*, *SQSTM1*, *TARDBP*, *TREM2*, *UBQLN2*, *VAPB* and *VCP*. Her symptoms progressed over time, including difficulty walking, difficulty swallowing and choking, tremors in the hands, voice changes and impulsivity. She died of aspiration pneumonia at the 71 years of age.

### Case 3 (mother of case 1)

This 72-year-old woman presented with shuffling gait, unsteadiness and frequent falls backwards at the age of 66. Her two brothers and their six children were all neurologically asymptomatic. She subsequently developed difficulty driving, struggled to complete activities of daily living, and she exhibited changes in her personality, characterized by increased social withdrawal and emotional volatility. She was diagnosed with PSP by a neurologist at age 69. As the disease progressed, she developed loss of muscle strength and swallowing difficulty. She was eventually admitted to a nursing home and required a feeding tube. Ultimately, she died of aspiration pneumonia at the age of 72.

## Materials and methods

### PET imaging

Case 1 underwent dynamic PET imaging following intravenous administration of 5.66 mCi [^18^F]APN-1607 on a Siemens HR + PET camera. Images were obtained from 0–90 and 120–180 min post-injection. Images were reconstructed in a 128 × 128 matrix (zoom = 2) with an iterative reconstruction algorithm (ordered subset expectation maximization), with random, attenuation and scatter corrections. Images were co-registered with 1 mm contiguous T1 MP-RAGE MRI images to delineate the anatomical regions of interest. Images are presented here as standardized uptake values from 60–90 min relative to a ventral cerebellar reference region (SUVr).

### Neuropathological examination

This study used brain tissue samples from nine cases, including eight control cases of typical CBD and typical PSP from the Mayo Clinic brain bank for neurodegenerative disorders. The summary of demographical and pathological features of the nine cases is provided in Additional file 1: Table S1.

The left hemibrain was fixed in formalin and embedded in paraffin. Paraffin-embedded 5-μm thick sections mounted on glass slides were stained with hematoxylin and eosin (H&E) and thioflavin S (Sigma-Aldrich, St. Louis, MO). Braak neurofibrillary tangle stage (NFT) and Thal amyloid phase were assigned by thioflavin S fluorescent microscopy according to published criteria as previously described [5, 22, 28, 32, 40]. Sections of the cortex, basal forebrain, striatum and brainstem were immunostained with antibodies against phosphorylated-tau (CP13; mouse monoclonal; 1:1000, gift from the late Dr. Peter Davies, Feinstein Institute, North Shore Hospital, NY), 3R tau (RD3, Millipore, Temecula, CA), 4R tau (RD4, Millipore), and phospshorylated-TDP-43 (pS409/410; mouse monoclonal; 1:5000; Cosmo Bio, Tokyo, Japan), phosphorylated-α-synuclein (EP1536Y; rabbit monoclonal; 1:40,000; Abcam, Waltham, MA), and amyloid β (6F/3D, mouse monoclonal; 1:250; Dako) using IHC Autostainer 480S (Thermo Fisher Scientific Inc., Waltham, MA) and DAKO EnVision™ + reagents (Dako, Carpinteria, CA). After immunostaining, the sections were counterstained with hematoxylin. Select sections were also stained with Gallyas silver staining. The slides were reviewed by two pathologists (SK and DWD). Our operational criteria for astrocytic lesions were as follows: Tufted astrocytes were characterized by thick, dense tau deposits in the proximal processes that typically radiated from the cell body in a sunburst or "tufted" pattern. Astrocytic plaques, on the other hand, were characterized by thin, radially oriented tau deposits in the distal processes surrounding a clear zone with the astrocytic cell body variable stained. Astrocytic lesions that did not fit into either tufted astrocytes or astrocytic plaques were categorized as “other” astrocytic lesions.

### Machine learning-based neuropathological diagnosis

To attempt to objectively classify the tau pathology in this patient, we implemented two machine learning-based diagnostic pipelines that we had previously developed [17, 20]. In both pipelines, digital images were used from tau-immunostained slides of the motor cortex, caudate nucleus and superior frontal gyrus.

The first pipeline integrates an object detection algorithm and a random forest classifier capable of distinguishing four tauopathies, namely Alzheimer's disease, CBD, Pick’s disease and PSP, based on tau immunohistochemistry of digital slide images [20]. We manually annotated a 5000 × 3000 pixel region of interest for each region of each case and applied the model to these images. Tau lesions (i.e. astrocytic plaques, coiled bodies, neuritic plaques, neuronal inclusions and tufted astrocytes) in each region were quantified, and the random forest classifier then assigned a tauopathy based on these values.

The second pipeline employs a clustering-constrained-attention multiple-instance learning (CLAM) model, which is a weakly-supervised learning algorithm. This model can classify five tauopathies (i.e., Alzheimer's disease, CBD, globular glial tauopathy, Pick’s disease and PSP) and non-tauopathy control cases [17]. In the present study, we used the model trained by a combined dataset of motor cortex, caudate nucleus and superior frontal gyrus, which showed the highest diagnostic accuracy in our previous study. This model predicted the diagnosis for each slide based upon whole slide images of tau-immunostained motor cortex, caudate nucleus and superior frontal gyrus.

### Western blotting

Western blotting was performed using the sarkosyl-insoluble fraction of tau from frozen brain tissues from motor cortex and superior frontal gyrus of Case 1, as well as control PSP and CBD cases, as previously described [18]. Insoluble tau samples were separated on 10% Tris–glycine gels (Thermo Fisher Scientific), transferred to nitrocellulose membranes (Millipore), and probed using the primary antibody against phosphorylated-tau (PHF1; mouse monoclonal; 1:1000; gift from the late Dr. Peter Davies, Feinstein Institute).

### 4R tau RT-QuIC

We performed a modified version of 4R tau RT-QuIC in 9 cases (Additional file 1: Table S1), including Case 1, as well as four PSP and four CBD cases, using frozen brain tissues from the motor cortex and superior frontal gyrus as previously described [36]. The distribution and severity of tau lesions of each PSP and CBD case are presented in Additional file 1: Tables S2 and S3. For this assay, a tau fragment comprising R1 – R4 and extending to residue 400 with cysteine to serine mutations, was utilized for amplification of brain-derived 4R tau aggregates. Cloning and expression followed procedures described previously [36]. Purification was modified slightly from prior tau RT-QuIC protocols; here, crude extract was heated at 70 °C following sonication, centrifuged and loaded onto a 20 ml CMFF HiTrap column. Protein was eluted over a 20 column volume (CV) gradient from 100–500 mM NaCl; fractions were pooled and loaded onto a 20 mL SPHP cation exchange column and eluted over a 40 CV gradient from 200–600 mM NaCl. Fractions were pooled and further purified and desalted by SEC chromatography on a 26/600 Superdex 200 column prior to lyophilization in 20 mM sodium phosphate buffer (pH 7.4). Lyophilized aliquots were resuspended in water prior to use. Protein preparations had a final concentration of 4 μM in 300 mM trisodium citrate buffered with 40 mM HEPES at pH 7.4, with 10 μM Thioflavin T (ThT) to track the formation of amyloid aggregates. The 10% brain homogenates that had been stored at −80 °C in PBS + protease inhibitor cocktail buffer were added to reactions at a final concentration of 1 × 10^–4^ brain homogenates. The reactions were split into individual wells of a 384-well optical bottom plate. Reactions were incubated with cyclical rounds of shaking and rest (500 rpm, orbital, 60 s on/60 s off) at 37 °C with periodic ThT fluorescence readings every 15 min.

### Genetic analyses

For genotyping and whole genome sequencing, genomic DNA was extracted from frozen cerebellum tissue of Case 1 using standard procedures. *MAPT* sequencing was performed in exons 7, 9, 10, 11, 12 and 13, as well as known pathogenic intronic mutations located at 50 bp on either side of each exon (e.g., IVS10 + 16 C>T) as previously described [23]. Genotyping for *MAPT* H1/H2 (SNP rs1052553 A/G, A = H1, G = H2) was assessed with TaqMan SNP genotyping assays (Applied Biosystems, Foster City, CA). Samples were assessed with whole genome sequencing by the Mayo Clinic Genome Analysis Core (https://www.mayo.edu/research/core-resources/genome-analysis-core/services/sequencing). Variant call files generated by the Mayo Clinic Bioinformatics Core were annotated for *MAPT* and *LRRK2* using Golden Helix SNP & Variation Suit v8.8.3.

## Results

### Neuropathological findings

**Fig. 2:**
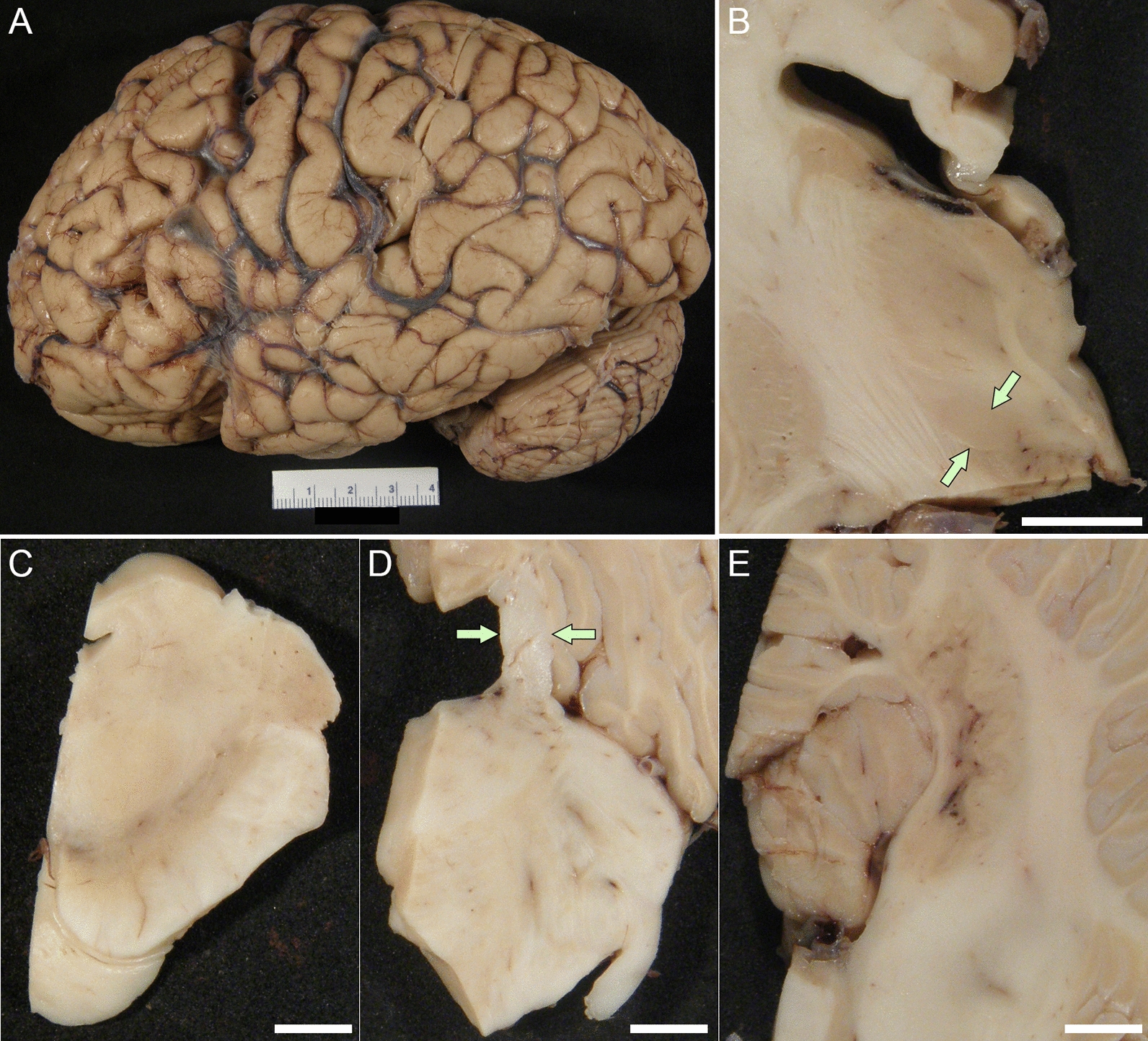
Macroscopic findings of Case 1. **A** The lateral view of the cerebral cortex is unremarkable. **B** The volume of subthalamic nucleus is preserved (arrows). **C** The melanin pigment is depleted in the substantia nigra. **D** The superior cerebellar peduncle is well preserved (arrows). **E** The dentate nucleus and cerebellar white matter are unremarkable. Scale bars: 1 cm in B, 5 mm in **C-E**

The fixed left hemibrain weighed 590 g. Macroscopic evaluation revealed mild cortical atrophy over the dorsolateral frontal lobe and the anterior temporal lobe, with focal enlargement of subarachnoid spaces (Fig. 2A). Sequential sections through the supratentorial tissues revealed mild enlargement of the frontal horn of the lateral ventricle. The hippocampal formation, amygdala, basal ganglia, thalamus and subthalamic nucleus (Fig. 2B) were unremarkable. The substantia nigra and the locus ceruleus had decreased pigmentation (Fig. 2C). The superior cerebellar peduncle was unremarkable (Fig. 2D). The cerebellar sections showed atrophy and gray discoloration of the hilus of the dentate nucleus (Fig. 2E).

**Fig. 3:**
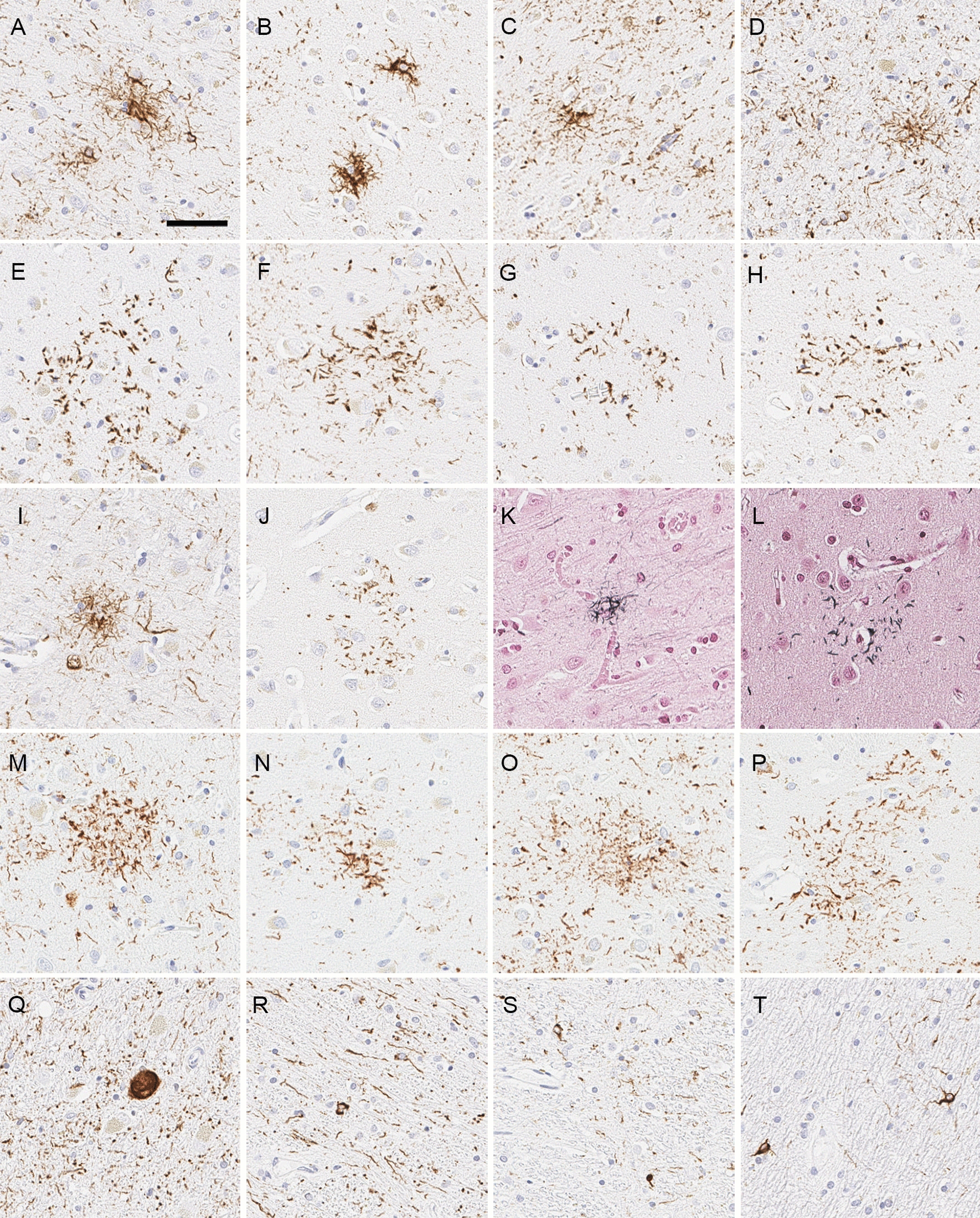
Representative images of histopathological findings of Case 1. **A-H** Immunohistochemistry for CP13 shows tufted astrocytes in the motor cortex (**A**), putamen (**B**), caudate nucleus (**C**), and midbrain tegmentum (**D**), as well as astrocytic plaques in the superior frontal gyrus (**E**), entorhinal cortex (**F**), occipitotemporal cortex (**G**), and putamen (**H**). **I**, **J** Immunohistochemistry for 4-repeat tau (RD4) reveals tufted astrocyte in the motor cortex (**I**) and astrocytic plaque in the superior frontal gyrus (**J**). **K**, **L** Gallyas silver staining shows tufted astrocyte in the motor cortex (**K**) and astrocytic plaque in the superior frontal gyrus (**L**). **N-P** There are astrocytic tau lesions that are categorized neither astrocytic plaques nor tufted astrocytes. **Q-T** Immunohistochemistry for CP13 shows globose tangles in the subthalamic nucleus (**M**); coiled bodies and threads in the thalamic fasciculus (**N**), red nucleus (**O**), and cerebellar white matter (**P**). Scale bar = 50 μm

The neocortex had a relatively unremarkable appearance on H&E, with only mild subjective neuronal loss and gliosis in the motor and premotor cortices. Immunohistochemistry for tau showed widespread neuronal and glial tau pathology in cortical and subcortical structures (Table 2). Tau immunohistochemistry revealed numerous tufted astrocytes in the motor cortex (Fig. 3A), putamen (Fig. 3B), caudate nucleus (Fig. 3C) and midbrain tegmentum (Fig. 3D). Astrocytic plaques were also observed in the superior frontal gyrus (Fig. 3E), entorhinal cortex (Fig. 3F), occipitotemporal cortex (Fig. 3G) and putamen (Fig. 3H). Tufted astrocytes and astrocytic plaques were both positive on 4R-tau immunohistochemistry (Fig. 3I-J) and with Gallyas silver staining (Fig. 3K-L). Additionally, there were atypical astrocytic lesions that were neither astrocytic plaques nor tufted astrocytes in these brain regions (Fig. 3M-P).

**Table 2:** The distribution and severity of tau lesions (Case 1)

Region	NFT & pre-NFT	Coiled bodies	Tufted astrocytes	Astrocytic plaques	Other astrocytic lesions	Tau^+^ threads
Temporal cortex	+++	+	−	++	+	++
Superior frontal gyrus	+++	++	+	+++	+	+++
Motor cortex	+++	++	+++	−	+	+++
Caudate/putamen	+++	++	+	+++	+	+++
Globus pallidus	++	++	−	−	−	++
Basal nucleus	++	+	−	−	−	+++
Hypothalamus	+++	+	−	−	−	+++
Ventral thalamus	+++	+	+	−	−	++
Subthalamic nucleus	+++	+	−	−	−	+++
Thalamic fasciculus	−	++	−	−	−	++
Red nucleus	+	++	−	−	−	++
Substantia nigra	++	+	−	−	−	+++
Oculomotor complex	++	+	−	−	+	++
Midbrain tectum	++	++	++	+	−	+++
Locus ceruleus	+++	−	−	−	−	+++
Pontine tegmentum	++	+	−	−	−	++
Pontine base	+	+	−	−	−	+
Medullary tegmentum	+++	+	−	−	−	+++
Inferior olivary nucleus	−	+	++	−	−	+
Dentate nucleus	++	−	−	−	−	+++
Cerebellar white matter	−	++	−	−	−	+

Some astrocytic tau lesions are hardly categorized into either tufted astrocyte or astrocytic plaques, which are categorized as other astrocytic lesions. *NFT* neurofibrillary tangle

The subthalamic nucleus had mild-to-moderate neuronal loss with many globose tangles and thread-like tau pathology (Fig. 3Q). The thalamic fasciculus had moderate threads and coiled bodies (Fig. 3R). The substantia nigra had mild-to-moderate neuronal loss with neuropil vacuolation and gliosis, which was marked in dorsal and medial cell groups. The red nucleus had minimal gliosis and moderate coiled bodies (Fig. 3S), but the midbrain tectum had more marked pathology. The locus ceruleus and pontine and medullary reticular formation had preserved neuronal populations, but many pretangles and threads. The lower brainstem was remarkable for only sparse pretangles and threads in the pontine base and in the inferior olivary nucleus. The cerebellum showed mild patchy Purkinje and internal granular cell loss. The cerebellar white matter had prominent threads and coiled bodies (Fig. 3T). The cerebellar dentate nucleus had minimal neuronal loss, but no grumose degeneration. The hilus and superior cerebellar peduncle showed mild myelinated fiber loss.

Thioflavin S fluorescence microscopy revealed a few NFTs in the subiculum and CA1 sector of the hippocampus, consistent with Braak NFT stage III. Sparse senile plaques were present in the superior temporal cortex and visual cortex but none in the middle frontal gyrus, inferior parietal gyrus, and motor cortex, consistent with Thal amyloid phase 2. We also performed immunohistochemistry for amyloid β to differentiate astrocytic plaques from senile plaques. There were no senile plaques in the caudate nucleus and only sparse diffuse plaques in the motor cortex and superior frontal gyrus and putamen, where astrocytic plaques or tufted astrocyte were frequent (Additional file 1: Fig. S1). Immunohistochemistry for phosphorylated-TDP-43 showed no neuronal or glial inclusions in the amygdala, hippocampus, cerebral cortices, striatum, or midbrain. Immunohistochemistry for α-synuclein did not reveal any Lewy-related pathology in the neocortices, amygdala, hippocampus, cingulate gyrus, basal ganglia, midbrain, pons, or medulla.

### Machine learning-based quantification analysis

Next, we assessed whether the two machine learning-based diagnostic pipelines could assist in classifying the tauopathy of this patient [17, 20]. We applied the object detection algorithm to three brain regions: the motor cortex, caudate nucleus, and superior frontal gyrus [20]. The quantitative results of each tau lesions are shown in Table 3, and the tau immunohistochemistry images after the analysis are given in Additional file 1: Figs. S2–S4. Based on these quantitative data, the random forest classifier diagnosed this patient as PSP.

**Table 3:** Quantitative assessment of tau lesions using the object detection model

	Tufted astrocyte	Astrocytic plaque	Coiled body	Neuronal inclusion	Neuritic plaque
Motor cortex	42	0	57	61	0
Caudate nucleus	20	50	5	86	0
Superior frontal gyrus	6	21	5	42	0

We also applied the CLAM-based pipeline to the whole slide images of these three brain regions [17]. The model predicted a diagnosis of PSP for both the motor cortex and superior frontal gyrus, but suggested CBD for the caudate nucleus. These results support the notion that the tauopathy of this patient showed mixed features of PSP and CBD, depending on the brain regions analyzed.

### Western blotting

**Fig. 4:**
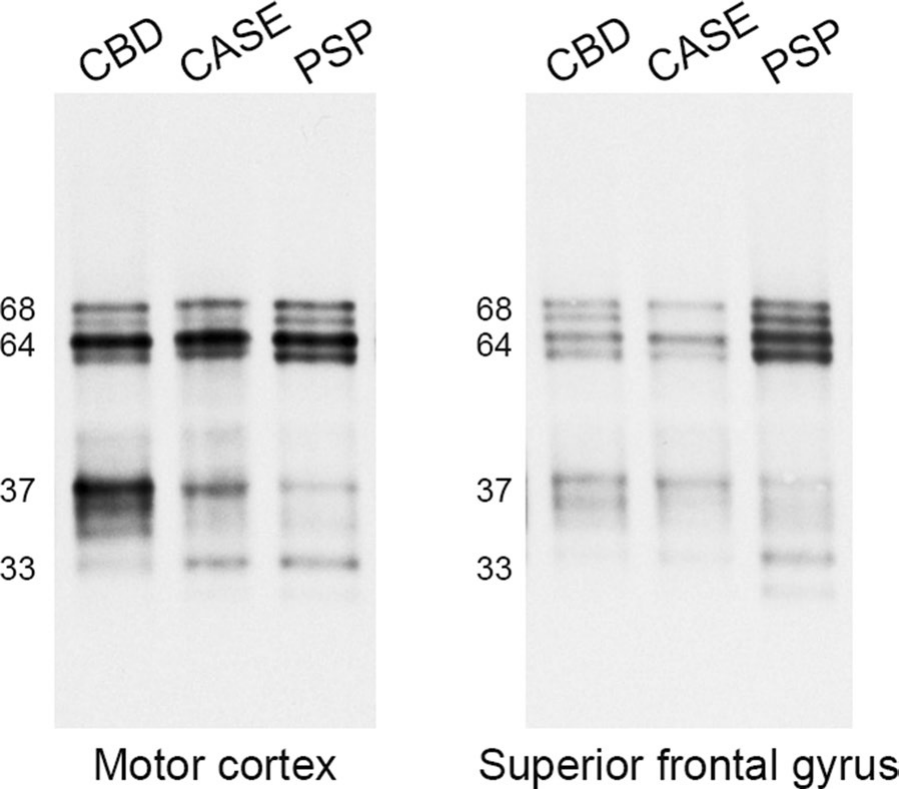
Western blotting of sarkosyl-insoluble tau. As with CBD and PSP, the index case (shown as CASE) has both 68 and 64 kDa bands in the motor cortex and superior frontal gyrus. As low molecular fragments, Case 1 has 37 and 33 kDa in the motor cortex and 37 kDa band in the superior frontal gyrus

To compare the biochemical features of insoluble tau Western blotting was performed on sarkosyl-insoluble tau from the motor cortex and superior frontal gyrus (Fig. 4). Two major bands at 68 and 64 kDa were observed, as well as controls cases of CBD and PSP. In CBD, there were two bands at approximately 37 kDa, and there was a band at approximately 33 kDa in PSP. The index case had bands at both 37 and 33 kDa in the motor cortex. In the superior frontal gyrus, the index case had only the 37 kDa band, consistent with CBD.

### RT-QuIC

**Fig. 5:**
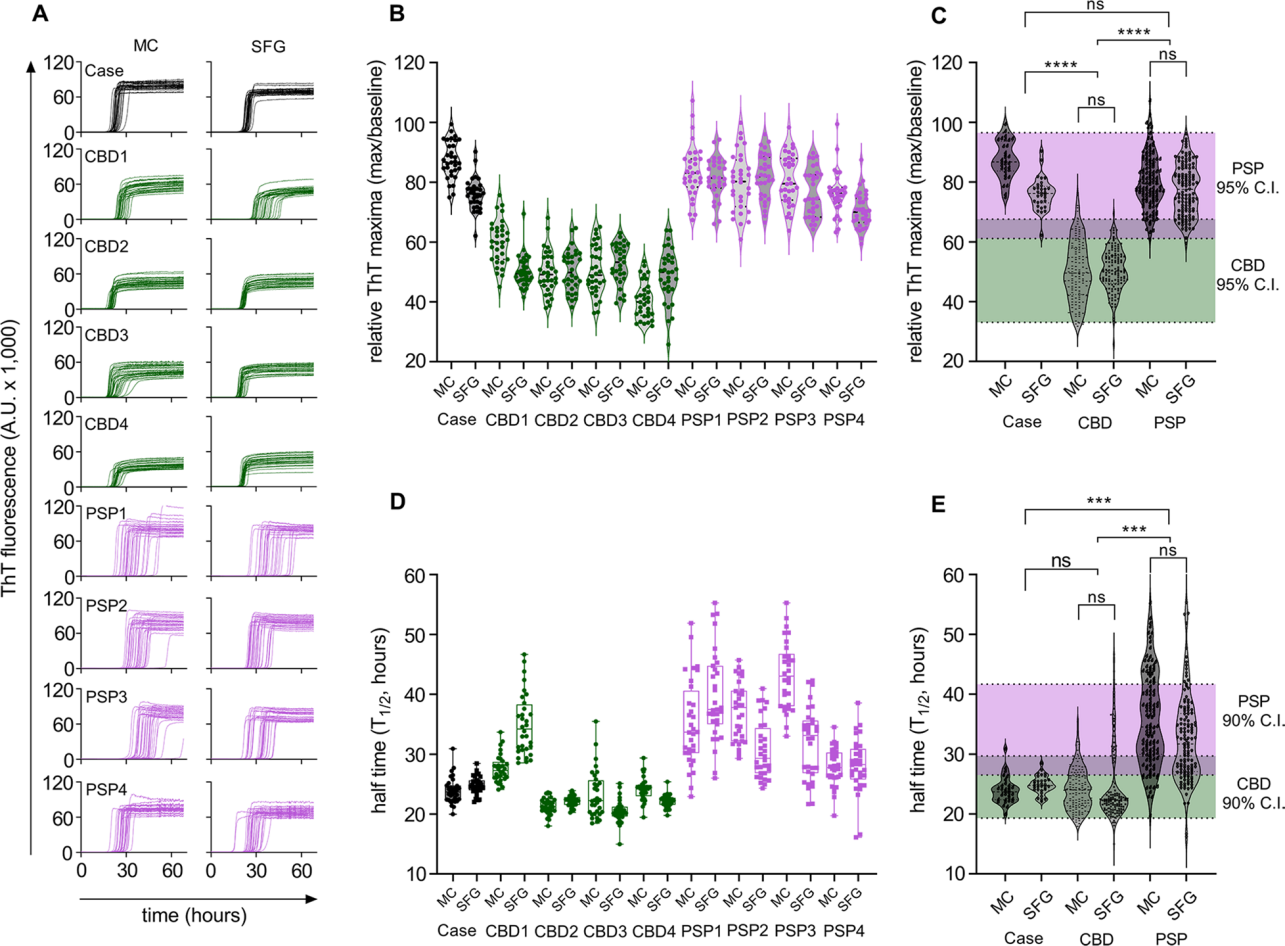
Tau RT-QuIC classification of Case 1. **A** Raw RT-QuIC traces of Case 1, CBD, PSP cases run in 32 replicates per indicated sample; MC, motor cortex; SFG, superior frontal gyrus. **B** Violin plots relative ThT maxima calculated by dividing peak ThT fluorescence values in panel A by baseline fluorescence values, points represent an individual trace from panel A. **C** Clustered peak ThT maxima analysis of Case 1 compared to known CBD and PSP cases; shaded areas represent 95% confidence intervals of all CBD cases (MC and SFG) versus all PSP cases (MC and SFG) by simple t-test; Asterisks represent *p* < 0.0001 by ordinary one-way ANOVA with multiple comparisons; ns, nonsignificant difference. **D** Half times of reactions in panel A calculated by simple sigmoidal dose–response fitting; boxes represent the interquartile range, whiskers represent outer quartiles (**E**) clustered T1/2 values of Case 1 compared to known CBD and PSP cases; shaded areas represent 90% confidence intervals of all CBD cases (MC and SFG) versus all PSP cases (MC and SFG) by simple t-test; Asterisks represent *p* < 0.001 by ordinary one-way ANOVA with multiple comparisons

We previously reported that tau RT-QuIC with brain homogenates successfully differentiated PSP from CBD [36]. ThT fluorescence amplitude can be used to differentiate between distinct conformers of recombinant 4R and 3R tau aggregates seeded by tauopathy brain homogenates [31, 36]. This method was adapted in this work to a singular 4R tau substrate to investigate if the present case had seeding activity analogous to that of PSP or CBD. Figure 5 shows (A) raw traces and (B-E) processed data and statistical analysis to sub-classify Case 1. Figure 5A depicts 32-replicate raw ThT fluorescence traces of K11 RT-QuIC reactions seeded with the motor cortex and superior frontal gyrus from Case 1 and 4 known CBD and PSP cases. Relative ThT fluorescence amplitudes of individual cases (B) and clustered cases (C). Aggregates formed by seeding with CBD, regardless of brain region, exhibited relative ThT fluorescence maxima spanning 33–67 RFU (95% CI, green shaded region in C). In contrast, aggregates seeded by PSP exhibited fluorescence maxima spanning 61–96 RFU (95% CI, purple shaded region in C). Fluorescence maxima of both motor cortex and superior frontal gyrus from Case 1 fell within the 95% CI of known PSP cases; 2 of 64 reactions from the presented case fell outside of this interval. Differences of means were analyzed by simple one-way ANOVA. ThT amplitudes of reactions seeded by controls (CBD < PSP) with extended recombinant 4R tau substrate are in agreement with previously-observed ThT amplitude clusters [36]. Figure 5D summarizes kinetics of reactions in panel A; points represent half times (T1/2) of reactions in hours, determined by fitting simple nonlinear regression models in GraphPad PRISM. Figure 5E similarly clusters T1/2 values by tauopathy and brain region. CBD-seeded reactions exhibited the fastest kinetics with T1/2 values spanning 18–29 h (90% CI, green shaded region in E), while PSP-seeded reactions exhibited more variability with T1/2 values spanning 27–42 h (90% CI, purple shaded region in E). As with analysis of variance of ThT maxima, T1/2 values were significantly different between CBD and PSP (*p* < 0.001). Reactions seeded by Case 1 exhibited T1/2 kinetics falling in the range of CBD-seeded reactions.

### Genetic analysis

Due to the strong family history of PSP, we suspected familial PSP due to *MAPT* mutations and performed sequencing of *MAPT* genes, but found no mutations. The patient was homozygous for the *MAPT* H1 haplotype. We next performed the whole genome sequencing to identify other potential genetic etiologies, but none were found in *MAPT* nor *LRRK2*. The only missense variant detected was LRRK2 p.M2397T, a common variant that is not considered pathogenic [27].

## Discussion

We report a patient with familial PSP with atypical neuropathological findings. The patient had neuronal and glial 4R tau pathology in the substantia nigra, subthalamic nucleus, globus pallidus, motor and premotor cortex, ventral thalamus, corpus striatum, and the olivopontocerebellar system, which is consistent with a diagnosis of PSP [8]. Depending upon the brain region, however, astrocytic lesions resembled either tufted astrocytes or astrocytic plaques; the former predominated in the motor cortex and midbrain tegmentum, while the latter was most frequent in the temporal, frontal and cingulate cortices. Additionally, the lack of atrophy in the subthalamic nucleus and superior cerebellar peduncle was atypical for PSP. Despite the patient presenting with Richardson syndrome and having strong family history of PSP, the neuropathological diagnosis was unclassified FTLD-tau, rather than PSP or CBD.

Given the complex and atypical nature of the neuropathological findings, we explored other approaches to understand the pathology in this patient. We used unbiased machine learning-based models that we previously developed [17, 20]. The first model, employing an object detection algorithm and random forest classifier [19]. This method classified the patient as PSP. This model, however, demonstrated certain limitations. First, it was trained to identify five predefined tau lesions, potentially resulting in misclassification or omission of atypical tau lesions in this patient. Furthermore, since all cases are forced into one of the four pre-defined tauopathies, this model may have difficulties with lesions that do not fit into the assigned categories.

To address these limitations, we utilized a second model based on a CLAM algorithm [17]. This model requires only slide-level labels, reducing the need for manual annotation of each tau lesion and mitigating potential subjectivity and labeling errors. Importantly, the diagnosis generated by this model is guided by the overall "impression" of the slide, rather a diagnosis based upon each individual lesion. This approach is particularly useful in cases with mixed or atypical tau lesions. Interestingly, when applied to our case, the diagnoses mirrored the mixed features observed in neuropathological examination, with PSP predicted for the motor cortex and superior frontal gyrus, but CBD predicted for the caudate nucleus.

To further characterize the tau pathology, we performed biochemical analyses. Immunoblotting of insoluble tau demonstrated 68 and 64 kDa bands, indicative of a 4R tauopathy. Tau from the superior frontal gyrus had a pattern consistent with CBD, reflecting abundant astrocytic plaques in this region. In contrast, tau from the motor cortex showed lower molecular weight bands at 37 and 33 kDa, consistent with mixed features of PSP and CBD [1]. Intriguingly, this banding pattern was also observed in a patient with unclassified 4R tauopathy reported by Nakano et al. [33]. Their patient presented with familial Parkinsonism without known mutations in *MAPT*, *DCTN1*, *PSEN1* or other young-onset Parkinsonism-related genes. The similarities with our patient support a diagnosis of an unclassified tauopathy.

As we previously reported, tau RT-QuIC using brain homogenates can effectively distinguish the seeding activity of tau protein of PSP and CBD using assay output parameters, such as fluorescence maxima and aggregate conformation [36]. Consistent with neuropathologic and biochemical analyses, tau RT-QuIC displayed characteristics of both PSP and CBD. The motor and frontal cortex samples resembled PSP in ThT maxima, which reflects the fluorescence levels achieved during the assay. Conversely, fibril amplification progressed at a rate similar to CBD, as shown by T_1/2_, representing fibril formation speed. Considering that amyloid formation kinetics are affected by seed conformer, reaction conditions and seed dose, it is interesting that seeding reactions had an amplitude similar to those of PSP, despite having equal or less insoluble tau protein on western blot, yet kinetics on the same scale as CBD-seeded reactions. These findings demonstrate characteristics of both PSP and CBD in the tau RT-QuIC assay, in line with the mixed pathology noted by histopathology and machine learning methods.

Although astrocytic plaques and tufted astrocytes are thought to be mutually exclusive, patients with both types of lesions have been reported [16, 39]. An autopsy study of a patient with a 5-year history of corticobasal syndrome was found to have 4R-tau pathology with features of both CBD and PSP [16]. In particular, there were both astrocytic plaques and tufted astrocytes. There was also neuronal loss and gliosis in the neocortex, subcortical nuclei, brainstem and cerebellum, which are features of CBD and PSP. Immunoblotting of sarkosyl-insoluble tau showed features of both CBD and PSP, including low molecular fragments at approximately 37- and 33-kDa. Another study reported two patients with CBD who had tufted astrocytes in addition to astrocytic plaques in the cerebral cortex [39]. Immunoblotting of sarkosyl-insoluble tau extracted from the frontal and parietal lobes showed low molecular fragments at approximately 37 kDa, consistent with the CBD. No mutations were found in the *MAPT* gene in either patient.

Compared to previously reported cases of coexistence of tufted astrocytes and astrocytic plaques, the unique aspect of the present case is the strong family history of PSP involving the patient’s mother and sister. While most PSP cases are sporadic, the *MAPT* gene mutations are the most common cause of familial PSP [10, 11]. We initially screened for mutations in the *MAPT* gene but found no pathological mutations in our proband. Forrest et al. reported three patients with PSP who had autosomal dominant PSP [10]. In one of these patients, *MAPT* gene sequencing revealed no mutations, suggesting the existence of unknown genetic causes beyond the *MAPT* gene.

While *LRRK2* is a monogenic causative gene for Parkinson’s disease, several mutations have also been suggested to be associated with PSP [3, 34, 35, 41]. Two patients with LRRK2 G2019S mutation had PSP-like neuronal and glial 4R tau pathology, but lacked neuronal loss in the subthalamic nucleus [34, 35]. In another patient from a family of autosomal dominant Parkinson’s disease due to LRRK2 p.R1441C, Parkinsonism and supranuclear gaze palsy were observed, and neuropathologic evaluation of this patient revealed PSP-like tau pathology, including globose tangles in the subthalamic nucleus and tufted astrocytes in the midbrain tectum [41]. Additionally, LRRK2 p.A1413T is a rare non-synonymous substitution found in screens of 1039 PSP patients, and it is also considered a potentially pathogenic LRRK2 variant [37]. In the present study, we detected only a common non-pathogenic LRRK2 substitution p.M2397T [27].

There are some limitations in our study. Given that our patient’s clinical symptoms were worse on the left side, the right hemibrain would be predicted to have more severe pathology; however, only the left side was evaluated. It is noteworthy that neuropathological evaluation was done using left hemibrain, and biochemical assays were performed using the right hemibrain; therefore, neuropathological findings did not necessarily reflect the results of Western blotting and RT-QuIC. Second, although the patient had a strong family history of PSP, neither her mother nor sister had pathologic confirmation. Detailed clinical information was also unavailable for review. Her sister underwent genetic analysis focusing on genes that cause amyotrophic lateral sclerosis and frontotemporal dementia. This suggests that her clinical presentation may have been atypical. DNA samples of the mother and sister of the patient were unavailable for study. Neuropathological and genetic assessments in the trio likely would have yielded a more definitive result.

In summary, we report a patient with familial PSP who did not carry mutations in *MAPT* or *LRRK2* genes. Our final neuropathological diagnosis is unclassified FTLD-tau, based on the coexistence of molecular and neuropathological features of both PSP and CBD. Despite utilizing both Western blotting and tau RT-QuIC analysis to clarify the tauopathy, this case could not be conclusively classified as PSP or CBD. This case emphasizes the potential complexity of FTLD-tau and the necessity for further research to better understand and classify such atypical presentations.

## Supplementary Information

**Additional file 1.** **Table 1**: Summary of pathological findings of cases used in RT-QuIC. Abbreviations: Braak, Braak NFT stage; F, female; M, male; Thal, Thal amyloid phase. **Table 2**: Semiquantitative assessment of tau lesions in four control CBD cases. C1 to C4 indicate CBD1 to CBD4, respectively. Abbreviations: NA, not assessed; NFT, neurofibrillary tangle. **Table 3**: Semiquantitative assessment of tau lesions in four control PSP cases. P1 to P4 indicate PSP1 to PSP4, respectively. Abbreviations: NFT, neurofibrillary tangle. **Figure 1**: Representative images of immunohistochemistry for tau and amyloid β in the motor cortex, caudate nucleus, and superior frontal gyrus. While tau pathologies are frequent (left column), only a few diffuse plaques are observed in amyloid β immunohistochemistry (right column).These findings indicate that these tau lesions do not contain amyloid β component. **Figure 2**: The result of object detection model in the motor cortex. CB (green) indicates coiled body, NI (purple) indicates neuronal inclusions, and TA (yellow) indicates tufted astrocyte. The numbers on the labels indicate the confidence score, which takes a number between 0 and 1. **Figure 3**: The result of object detection model in the caudate nucleus. AP (red) indicates astrocytic plaques, CB (green) indicates coiled body, NI (purple) indicates neuronal inclusions, and TA (yellow) indicates tufted astrocyte. The numbers on the labels indicate the confidence score, which takes a number between 0 and 1. **Figure 4**: The result of object detection model in the caudate nucleus. AP (red) indicates astrocyticplaques, CB (green) indicates coiled body, NI (purple) indicates neuronal inclusions, and TA (yellow) indicates tufted astrocyte. The numbers on the labels indicate the confidence score, which takes a number between 0 and 1.

## Data Availability

The data that support the findings of this study are available from the corresponding author upon reasonable request.

## Abbreviations

3R: 3-Repeat
4R: 4-Repeat
CBD: Corticobasal degeneration
CLAM: Clustering-constrained-attention multiple-instance learning
FTLD: Frontotemporal lobar degeneration
H&E: Hematoxylin and eosin
NFT: Neurofibrillary tangle stage
PET: Positron emission tomography
PSP: Progressive supranuclear palsy
RT-QuIC: Real-time quaking-induced conversion
ThT: Thioflavin T
