# Supplementary material for: Case report of a patient with unclassified tauopathy with molecular and neuropathological features of both progressive supranuclear palsy and corticobasal degeneration
Source: Acta Neuropathol Commun. 2023 Jun 1;11:88. doi: 10.1186/s40478-023-01584-z (PMC10236843; doi:10.1186/s40478-023-01584-z)
Supplement: Supplementary file 1 — Additional file 1. Table 1: Summary of pathological findings of cases used in RT-QuIC. Abbreviations: Braak, Braak NFT stage; F, female; M, male; Thal, Thal amyloid phase. Table 2: Semiquantitative assessment of tau lesions in four control CBD cases. C1 to C4 indicate CBD1 to CBD4, respectively. Abbreviations: NA, not assessed; NFT, neurofibrillary tangle. Table 3: Semiquantitative assessment of tau lesions in four control PSP cases. P1 to P4 indicate PSP1 to PSP4, respectively. Abbreviations: NFT, neurofibrillary tangle. Figure 1: Representative images of immunohistochemistry for tau and amyloid β in the motor cortex, caudate nucleus, and superior frontal gyrus. While tau pathologies are frequent (left column), only a few diffuse plaques are observed in amyloid β immunohistochemistry (right column).These findings indicate that these tau lesions do not contain amyloid β component. Figure 2: The result of object detection model in the motor cortex. CB (green) indicates coiled body, NI (purple) indicates neuronal inclusions, and TA (yellow) indicates tufted astrocyte. The numbers on the labels indicate the confidence score, which takes a number between 0 and 1. Figure 3: The result of object detection model in the caudate nucleus. AP (red) indicates astrocytic plaques, CB (green) indicates coiled body, NI (purple) indicates neuronal inclusions, and TA (yellow) indicates tufted astrocyte. The numbers on the labels indicate the confidence score, which takes a number between 0 and 1. Figure 4: The result of object detection model in the caudate nucleus. AP (red) indicates astrocyticplaques, CB (green) indicates coiled body, NI (purple) indicates neuronal inclusions, and TA (yellow) indicates tufted astrocyte. The numbers on the labels indicate the confidence score, which takes a number between 0 and 1. [file 40478_2023_1584_MOESM1_ESM.docx]

**Supplementary Table S1**: Summary of pathological findings of cases used in RT-QuIC.

| **Case #** | **Pathological diagnosis** | **Age** | **Sex** | **Braak** | **Thal** |
| --- | --- | --- | --- | --- | --- |
| Index Case | PSP | 75 | F | III | 2 |
| CBD1 | CBD | 80 | F | II | 2 |
| CBD2 | CBD | 62 | M | II | 2 |
| CBD3 | CBD | 72 | M | III | 1 |
| CBD4 | CBD | 70 | M | II | 2 |
| PSP1 | PSP | 76 | F | I | 2 |
| PSP2 | PSP | 67 | F | II | 1 |
| PSP3 | PSP | 67 | M | II | 1 |
| PSP4 | PSP | 75 | M | 0 | 2 |

Abbreviations: Braak, Braak NFT stage; F, female; M, male; Thal, Thal amyloid phase.

**Supplementary Table S2**: Semiquantitative assessment of tau lesions in four control CBD cases.

| **Region** | **NFT & pre-NFT** | | | | **Coiled bodies** | | | | **Astrocytic plaques** | | | | **Tau^+^ threads** | | | |
| --- | --- | --- | --- | --- | --- | --- | --- | --- | --- | --- | --- | --- | --- | --- | --- | --- |
|  | C1 | C2 | C3 | C4 | C1 | C2 | C3 | C4 | C1 | C2 | C3 | C4 | C1 | C2 | C3 | C4 |
| *Temporal cortex* | + | ++ | + | ++ | - | + | - | + | + | + | - | + | - | ++ | - | + |
| *Superior frontal gyrus* | +++ | +++ | +++ | +++ | + | +++ | ++ | ++ | ++ | +++ | ++ | +++ | ++ | +++ | +++ | +++ |
| *Motor cortex* | +++ | +++ | +++ | + | ++ | ++ | ++ | + | ++ | + | + | + | +++ | +++ | +++ | + |
| *Caudate/putamen* | ++ | +++ | ++ | +++ | + | ++ | + | +++ | ++ | +++ | +++ | +++ | ++ | +++ | ++ | +++ |
| *Globus pallidus* | + | ++ | ++ | +++ | - | ++ | ++ | - | - | - | - | - | + | +++ | +++ | +++ |
| *Basal nucleus* | +++ | +++ | +++ | +++ | - | + | - | + | - | - | - | -- | ++ | +++ | +++ | +++ |
| *Hypothalamus* | + | ++ | ++ | +++ | - | - | - | - | - | - | - | - | + | + | ++ | ++ |
| *Ventral thalamus* | + | +++ | +++ | +++ | - | + | - | + | - | ++ | - | - | + | +++ | +++ | +++ |
| *Subthalamic nucleus* | + | +++ | +++ | +++ | - | + | - | + | - | - | - | - | ++ | +++ | +++ | +++ |
| *Thalamic fasciculus* | - | - | - | - | - | ++ | + | + | - | - | - | - | ++ | +++ | ++ | +++ |
| *Red nucleus* | + | ++ | + | + |  | ++ | - | + | - | - | - | - | - | +++ | + | + |
| *Substantia nigra* | + | +++ | +++ | +++ | - | + | + | + | - | - | - | - | + | +++ | +++ | +++ |
| *Oculomotor complex* | + | ++ | +++ | ++ | - | - | - | - | - | - | - | - | - | +++ | +++ | ++ |
| *Midbrain tectum* | + | ++ | +++ | +++ | - | + | + | + | - | - | ++ | ++ | ++ | +++ | +++ | +++ |
| *Locus ceruleus* | + | +++ | NA | +++ | - | + | NA | - | - | - | NA | - | + | +++ | NA | ++ |
| *Pontine tegmentum* | ++ | +++ | + | +++ | - | + | - | + | - | - | - | - | ++ | +++ | ++ | +++ |
| *Pontine base* | - | +++ | + | ++ | - | + | + | + | - | - | - | - | + | ++ | + | ++ |
| *Medullary tegmentum* | + | +++ | +++ | +++ | - | + | - | + | - | - | - | - | + | +++ | +++ | +++ |
| *Inferior olive* | - | - | ++ | + | - | + | - | - | - | - | - | - | + | ++ | ++ | ++ |
| *Dentate nucleus* | - | +++ | +++ | + | - | + | - | - | - | - | - | - | + | ++ | ++ | ++ |
| *Cerebellar white matter* | - | - | - | - | - | + | - | + | - | - | - | - | - | + | + | + |

C1 to C4 indicate CBD1 to CBD4, respectively. Abbreviations: NA, not assessed; NFT, neurofibrillary tangle.

**Supplementary Table S3**: Semiquantitative assessment of tau lesions in four control PSP cases.

| **Region** | **NFT & pre-NFT** | | | | **Coiled bodies** | | | | **Tufted astrocytes** | | | | **Tau^+^ threads** | | | |
| --- | --- | --- | --- | --- | --- | --- | --- | --- | --- | --- | --- | --- | --- | --- | --- | --- |
|  | P1 | P2 | P3 | P4 | P1 | P2 | P3 | P4 | P1 | P2 | P3 | P4 | P1 | P2 | P3 | P4 |
| *Temporal cortex* | + | + | - | + | - | + | - | + | - | - | + | + | - | - | - | - |
| *Superior frontal gyrus* | ++ | + | ++ | ++ | + | ++ | + | +++ | ++ | ++ | ++ | +++ | - | + | + | + |
| *Motor cortex* | +++ | + | ++ | +++ | ++ | +++ | ++ | +++ | +++ | ++ | +++ | +++ | ++ | ++ | ++ | +++ |
| *Caudate/putamen* | ++ | ++ | ++ | ++ | + | +++ | +++ | +++ | +++ | +++ | +++ | +++ | + | ++ | + | + |
| *Globus pallidus* | ++ | +++ | ++ | ++ | ++ | +++ | ++ | +++ | - | + | + | + | ++ | +++ | ++ | +++ |
| *Basal nucleus* | +++ | +++ | +++ | +++ | + | ++ | + | + | - | + | + | - | ++ | +++ | +++ | ++ |
| *Hypothalamus* | ++ | +++ | +++ | ++ | - | - | - | - | - | - | - | - | + | ++ | ++ | + |
| *Ventral thalamus* | ++ | +++ | ++ | ++ | ++ | +++ | +++ | +++ | + | + | + | ++ | +++ | +++ | +++ | +++ |
| *Subthalamic nucleus* | ++ | +++ | +++ | +++ | ++ | +++ | ++ | +++ | + | - | + | + | ++ | +++ | +++ | +++ |
| *Thalamic fasciculus* | - | - | - | - | + | +++ | +++ | +++ | - | - | - | - | +++ | ++ | +++ | +++ |
| *Red nucleus* | ++ | ++ | ++ | ++ | +++ | +++ | +++ | ++ | ++ | + | + | - | +++ | +++ | +++ | +++ |
| *Substantia nigra* | +++ | +++ | ++ | ++ | + | ++ | ++ | ++ | - | + | ++ | - | +++ | +++ | ++ | +++ |
| *Oculomotor complex* | +++ | +++ | +++ | ++ | - | + | - | - | - | - | - | - | +++ | ++ | +++ | +++ |
| *Midbrain tectum* | +++ | +++ | ++ | +++ | +++ | +++ | ++ | +++ | +++ | +++ | + | ++ | ++ | +++ | +++ | +++ |
| *Locus ceruleus* | +++ | +++ | +++ | +++ | - | - | - | - | - | - | - | - | +++ | +++ | +++ | +++ |
| *Pontine tegmentum* | +++ | +++ | +++ | ++ | +++ | +++ | ++ | +++ | - | - | - | - | +++ | +++ | +++ | +++ |
| *Pontine base* | +++ | +++ | +++ | +++ | + | + | ++ | ++ | - | - | - | - | +++ | ++ | +++ | ++ |
| *Medullary tegmentum* | + | +++ | +++ | ++ | + | +++ | ++ | + | - | - | - | - | ++ | +++ | +++ | +++ |
| *Inferior olive* | +++ | +++ | + | + | - | ++ | + | ++ | + | + | + | - | +++ | +++ | +++ | +++ |
| *Dentate nucleus* | - | ++ | ++ | ++ | + | + | + | ++ | - | - | + | - | + | ++ | ++ | ++ |
| *Cerebellar white matter* | + | - | - | - | - | ++ | +++ | +++ | - | - | - | - | - | ++ | +++ | ++ |

P1 to P4 indicate PSP1 to PSP4, respectively. Abbreviations: NFT, neurofibrillary tangle.


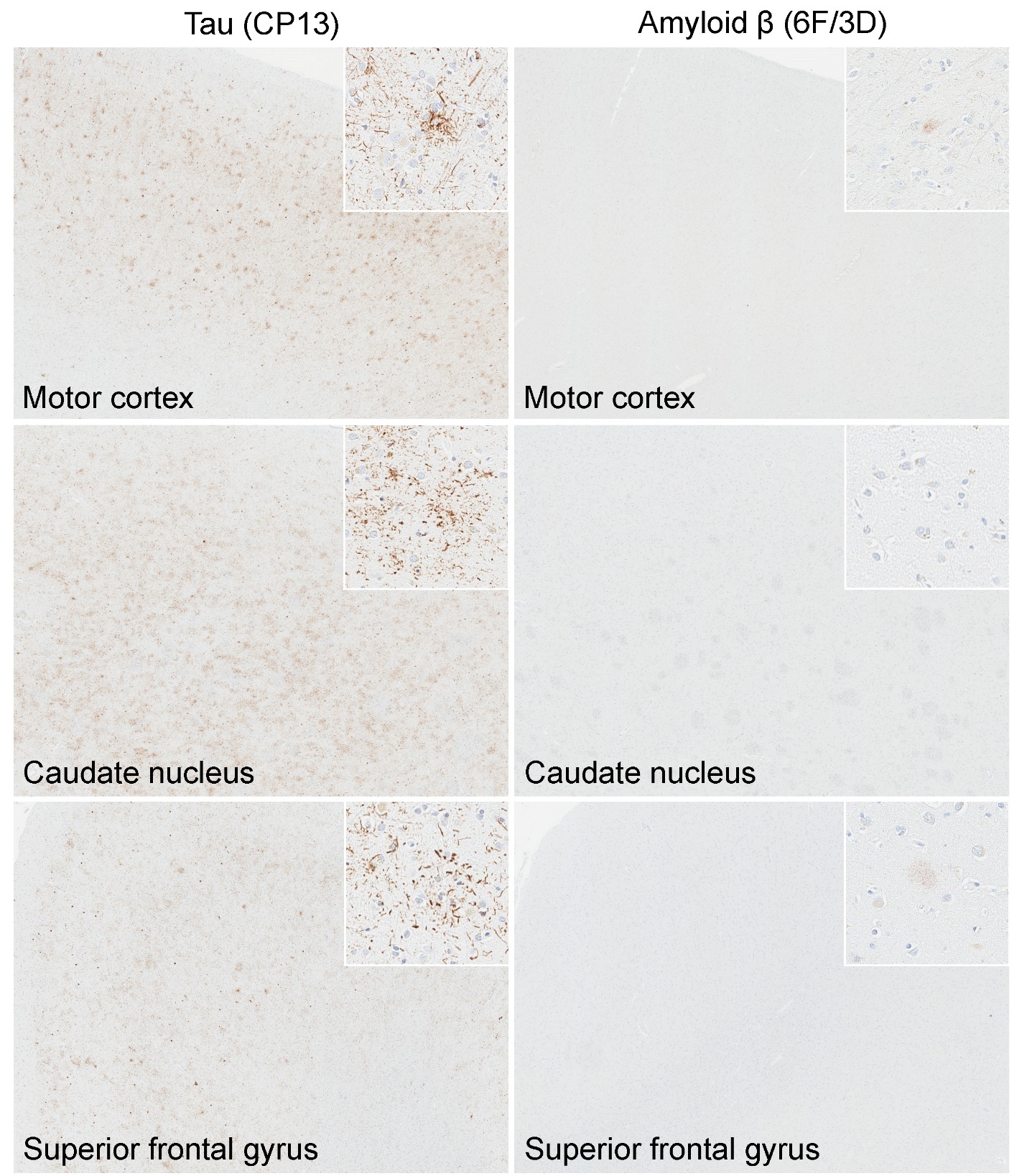


**Supplementary Figure S1**: Representative images of immunohistochemistry for tau and amyloid β in the motor cortex, caudate nucleus, and superior frontal gyrus. While tau pathologies are frequent (left column), only a few diffuse plaques are observed in amyloid β immunohistochemistry (right column).These findings indicate that these tau lesions do not contain amyloid β component.


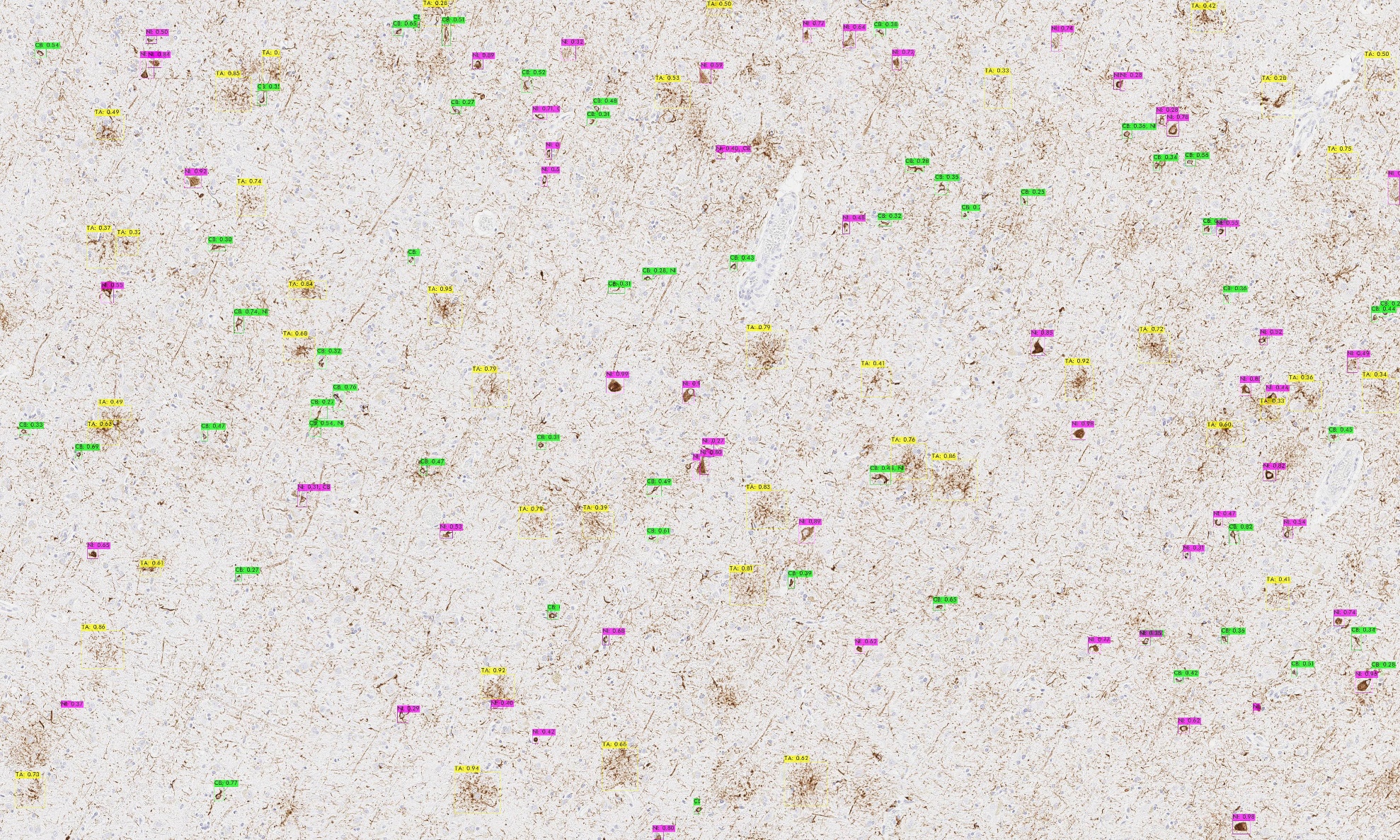


**Supplementary Figure S2**: The result of object detection model in the motor cortex. CB (green) indicates coiled body, NI (purple) indicates neuronal inclusions, and TA (yellow) indicates tufted astrocyte. The numbers on the labels indicate the confidence score, which takes a number between 0 and 1.


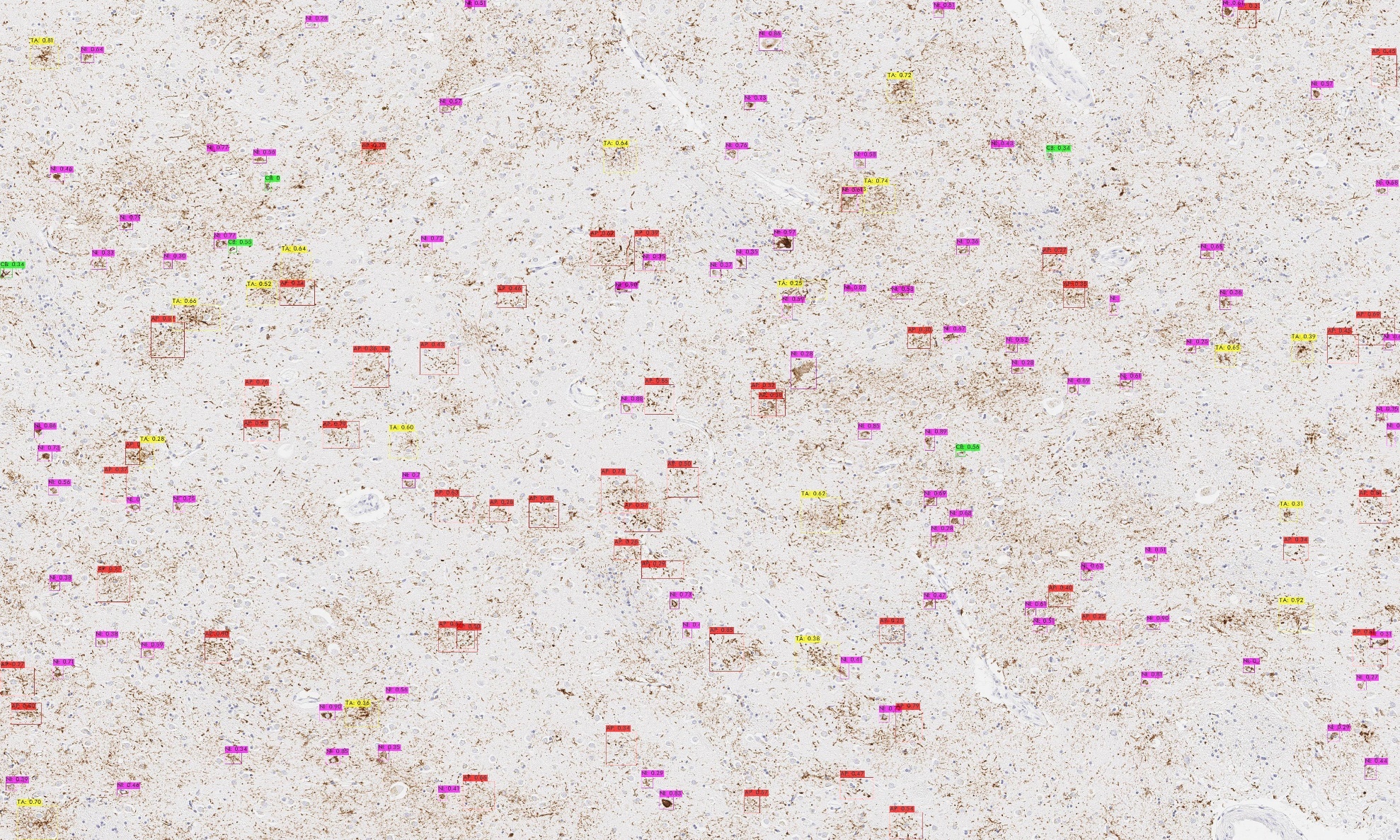


**Supplementary Figure S3**: The result of object detection model in the caudate nucleus. AP (red) indicates astrocytic plaques, CB (green) indicates coiled body, NI (purple) indicates neuronal inclusions, and TA (yellow) indicates tufted astrocyte. The numbers on the labels indicate the confidence score, which takes a number between 0 and 1.


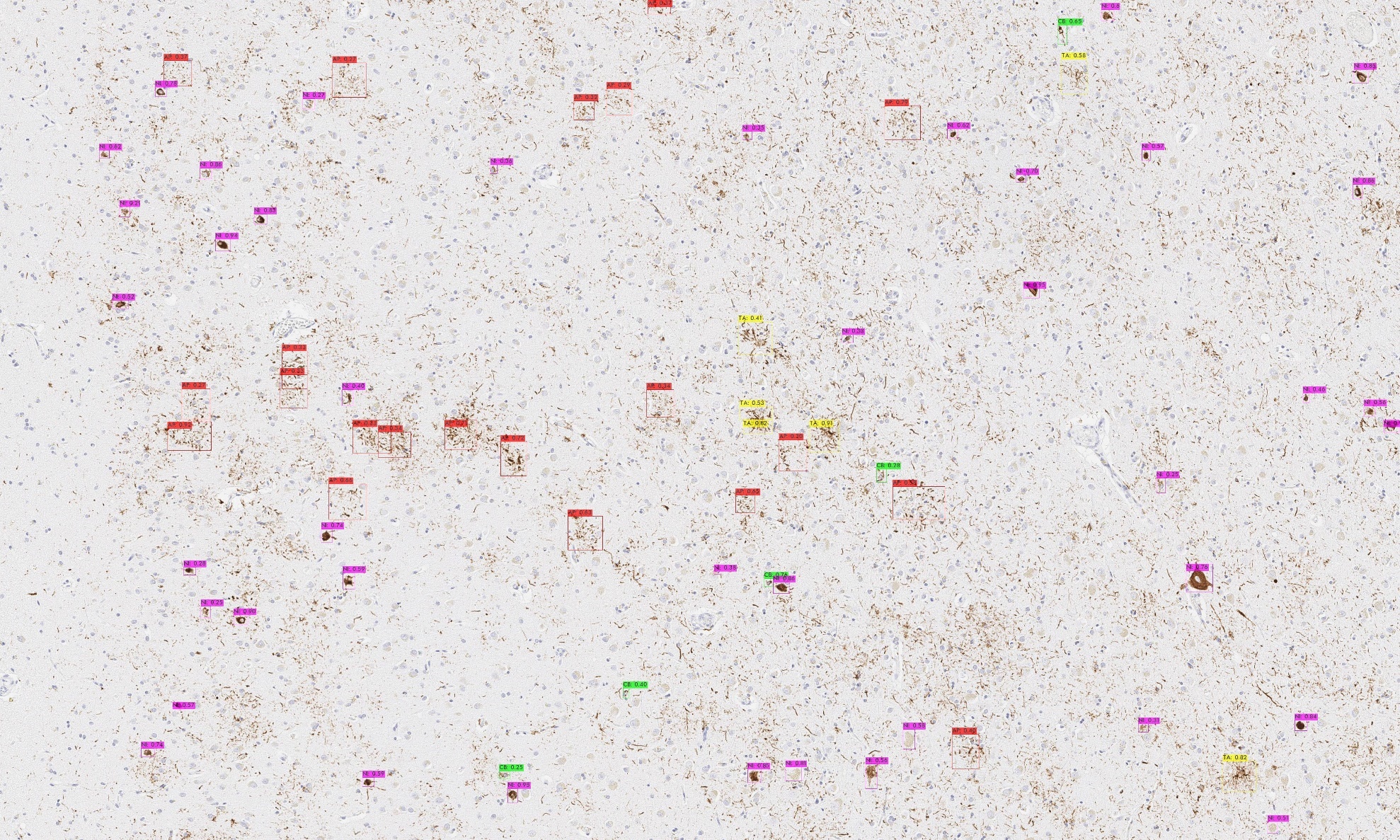


**Supplementary Figure S4**: The result of object detection model in the caudate nucleus. AP (red) indicates astrocytic plaques, CB (green) indicates coiled body, NI (purple) indicates neuronal inclusions, and TA (yellow) indicates tufted astrocyte. The numbers on the labels indicate the confidence score, which takes a number between 0 and 1.
